# ZHENG-Omics Application in ZHENG Classification and Treatment: Chinese Personalized Medicine

**DOI:** 10.1155/2013/235969

**Published:** 2013-04-03

**Authors:** Jianye Dai, Junwei Fang, Shujun Sun, Qiwen Chen, Huijuan Cao, Ningning Zheng, Yongyu Zhang, Aiping Lu

**Affiliations:** ^1^Center for Traditional Chinese Medicine and Systems Biology, Shanghai University of Traditional Chinese Medicine, Shanghai 201203, China; ^2^Department of Integrated Oncology, Shanghai Cancer Center, Fudan University, Shanghai 200032, China; ^3^School of Chinese Medicine, Hong Kong Baptist University, Hong Kong

## Abstract

With the hope to provide an effective approach for personalized diagnosis and treatment clinically, traditional chinese medicine (TCM) is being paid increasing attention as a complementary and alternative medicine. It performs treatment based on ZHENG (TCM syndrome) classification, which could be identified clinical special phenotypes by symptoms and signs of patients even if they have a different disease. However, it caused controversy because ZHENG classification only depends on observation, knowledge, and clinical experience of TCM practitioners, which lacks objectivity and repeatability. Although researchers and scientists of TCM have done some work with a lot of beneficial methods, the results could not reach satisfactory with the shortcomings of generalizing the entire state of the body or ignoring the patients' feelings. By total summary, mining, and integration of existing researches, the present paper attempts to introduce a novel macro-microconcept of ZHENG-omics, with the prospect of bright future in providing an objective and repeatable approach for Chinese personalized medicine in an effective way. In this paper, we give the brief introduction and preliminary validation, and discuss strategies and system-oriented technologies for achieving this goal.

## 1. Introduction

After the accomplishment of the Human Genome Project, personalized medicine is looming in the horizon. Recognizing continuous genetic variation and expensive cost, it seems that it would be difficult to perform personalized drug therapy for a wide range of major diseases using genomic knowledge alone, although it is clearly important [1]. Alternatively, a holistic approach attempting to bring the body, mind, and spirit into harmony [2, 3], traditional chinese medicine (TCM) combined with the principle of “ZHENG (TCM Syndrome) Classification and Treatment” (ZCT, *bian zheng lun zhi*, in Chinese), may bring personalized medicine to light in an efficient way. Researches have been conducted by He et al. [4] and Lu et al. [5] to illustrate that the effective rate of treatment based on ZHENG classification may improve the specificity and efficiency in both TCM and Western medicine. The success of personalized medicine relies on having accurate diagnostic tests that identify patients who can benefit from targeted therapies [6]. While in TCM, ZHENG classification is performed by four diagnostic methods: looking, listening and smelling, asking, and touching. Still it is argued, as it depended on clinical observation and TCM practitioner's experience, which would be subjective and unrepeatable. So, a great breakthrough in TCM diagnosis with objectivity, repeatability, and comprehensiveness is needed.

Actually, researchers and scientists of TCM have done some work with a lot of beneficial methods, such as physiology and biochemistry [7], molecular biology [8], and tongue image digitization [9, 10], to classify ZHENGs and evaluate the treatment based on ZHENG classification. Though the researches are beneficial, they are far from satisfactory. The main reason accounting for the results may be that these methods only focus on one or several indicators, which cannot generalize the entire state of the body. However, with the advent of the era of systems biology a broader technology platform was established for the study of TCM. The omic technologies provide integral, systemic, and dynamic technology platforms and lay the foundation for the systemic study at a higher level. The system point of view provides us with an important idea to sweep aside the fog of mysterious TCM and may be a breakthrough point of incorporating Eastern medicine with Western medicine. Though the researches based on system biology have had great progresses recently, there is a little flaw of ignoring patients' feelings in the only omic data.

So, through existing researches of system biology application in ZCT, we would like to propose further a new macro-microconcept, namely, “ZHENG-Omics,” which is defined as “a systematic approach for targeting individual patient, guiding treatment, and predicting the outcome of personalized treatment by global NET-Markers (combination of genes, proteins, metabolites, symptoms and others based on the mathematical model), on the basis of ZCT”. In this paper, after comprehensive reviewed recent researches, we will gradually show you brief introduction and preliminary validation and discuss strategies and system-oriented technologies for achieving the goal of providing an objective and repeatable approach for Chinese personalized medicine in an effective way.

## 2. Research Status of System Biology Application in ZCT

TCM researchers have attempted some beneficial works. Research in the area of Yin-Yang sets a good example. Yin-Yang (two opposite, complementary, interdependent, and exchangeable aspects of nature), the general principles in eight, are used to categorize natural phenomena, which helped us understand, prevent, and cure disease. The classification of Yin-Yang usually is the first step in the ZHENG classification. Systems biology was proved to be a useful tool for the study of Yin-Yang, although the concept of Yin-Yang is abstract, mysterious, and obscure. So far the most researches of Yin-Yang syndrome are concentrated on Yin-deficiency syndrome and Yang-deficiency syndrome. Genomic information showed that Yang-deficiency patients had significant difference in Haplotype 25 (Hap25) of APM1 rs7627128, rs1063539, PSMB7, and CXCR4 compared to control group; however, Haplotype13 of PPARG in Yin-deficiency patients [11, 12]. At the proteomic level, it was found that CRP, CRH, IL10, ACE, PTH, MPO, CRH, PTH, PRL, BRCA1, BRCA2 [13], transthyretin, plasma retinol-binding protein precursor and chain A-prealbumin [14], liver protein, and protein of the liver mitochondria [15] levels correlated with Yang-deficiency syndrome. It is especially worth noting that metabonomics is widely and deeply utilized in Yin-Yang classification. Wang et al. [16] and Lu et al. [17] have, respectively, found different metabolites between Kidney-Yin and Kidney-Yang deficiency syndrome, which are estrone, creatinine, uric acid, indoxyl sulfate and so on, demonstrating the notable differences of these two ZHENGs. From these previous researches we realized that it is possible to establish a group of preliminary global net-markers to identify the Yin-Yang deficiency syndrome. Though there has been no research on Net-Markers of Yin-Yang syndrome, the combination of network biology and Bioinformatics has been used by Li et al. to investigate novel biomarkers for cold-heat syndrome which partly represents the Yin-Yang syndrome in TCM [18, 19]. 

However, ZHENG classification is not the ultimate goal, but a progression towards treatment and curing of diseases. Unfortunately, current evaluation methods are conducted ignoring theories of TCM and are unable to bring this traditional medicine which has been validated in clinical practice to light. This may cripple public confidence in the effectiveness of TCM. To counter this, an objective method on the basis of holistic TCM must be developed. What is gratifying systems biology, especially metabonomics, has shown promising application prospect in its practice. For example, it was demonstrated in our previous experiment that if different ZHENGs of hepatitis-B-caused cirrhosis patients were treated by the same therapy, they would show various responses [20]. Similar research has been performed by Chen et al. [21] to study the biochemical profiles of hydrocortisone-induced animal models, which evaluated the effectiveness of *Herba Cistanches Deserticolae* that is formulated to warm and reinforce kidney Yang to intervene Kidney-Yang deficiency rats. A good correlation between the chemical profile and the progress of treatment was observed. In addition to this study, Wang et al. [16] and Lu et al. [17] evaluated the effectiveness of* Liuwei Dihuang Pill* and *Rhizoma Drynariae,* respectively, in Kidney-Yin and Kidney-Yang syndromes and satisfactory results were obtained as well.

Due to space limitation and insufficient research, other research has not been touched upon. To systematically note ZHENG research as being performed presently, clinical and experimental studies have been summarized in Tables 1 and 2, with respect to the combination of ZHENG in TCM and disease in western medicine.

## 3. Brief Introduction of the Origin of “ZHENG-Omics”

Our thought is not groundless but derived from former researches, although most of which were discrete and without a systemic and consistent research strategy. ZHENG-Omics is not simple combination of systems biology and ZCT. Their association could play bigger roles in helping mutually to remedy deficiencies. One, the systems biology research and philosophy of TCM coincide in many ways. They share similar attributes in many aspects, especially in a holistic approach. Two, the strategy employing a dynamic noninvasive approach is very important for the acquisition of long-term, large-scale samples. The thorough knowledge of ZHENGs' connotation and intervention may evolve to a dynamic classification approach. Three, a nontargeting concept may provide freedom for exploration of unknown classifications of disease states without being bound by the existing methods familiar to Western medicine. Finally, digital results can be integrated for ZHENG classification through statistical analysis and database building and modeling. Most importantly, digitalization of information can be absorbed from scholars without a TCM background into this field of research.

The scope of ZHENG-omics can be illustrated as follows. Firstly, clinical symptoms are classified into several groups according to the principle of ZHENG classification and the difference in biological markers, including DNA, RNA, proteins, and metabolites, are identified among these groups. Secondly, by integrating of genes, proteins, metabolites, symptoms, and others, NET-Markers of ZHENG will be obtained from former differences by bioinformatics and other mathematical analysis. The markers could then be used to provide the basis in developing a possible population-screening tool for selecting target individuals and creating evaluation index for personalized treatment based on ZHENG classification. Finally, ZHENG-Omics will give an objective and practical evaluation to the classical ZCT.

## 4. Preliminary Validation

**Figure 1:**
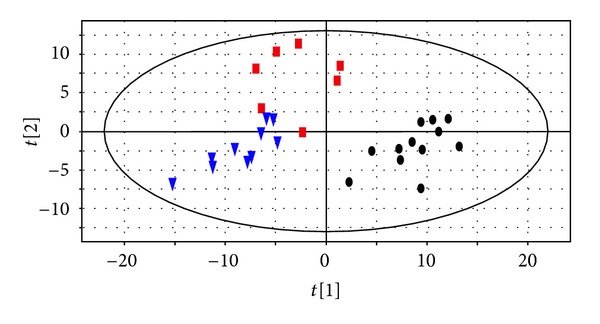
PLS-DA score plots between healthy subjects and Hepatitis B caused Cirrhosis subjects. Black dots, Red boxes, blue triangles refer to healthy subjects, Liver-Gallbladder Dampness-Heat Syndrome and Liver-Kidney Yin Deficiency Syndrome of Hepatitis B caused Cirrhosis subjects, respectively.

To explore the feasibility of our thought process, a preliminary case study was performed. The study was performed in accordance with the principles contained in the Declaration of Helsinki and was approved by the local ethics committee. Urine samples from 12 healthy volunteers (control group, CG) and 17 patients (Hepatitis-B-caused cirrhosis group, HBCG) were analyzed by gas chromatography mass spectrometry (GC/MS), and multivariate statistical analysis was performed by Simca-P 12.0 Software package (Umetrics, Umea, Sweden) [68]. Though the patients were treated by Fuzheng Huayu tablets (FZHY tablets, Chinese patent medicine) in the same way as lacking significant deviation in Child-Pugh Score, they were classified into 2 ZHENG types by the TCM practitioner according to the recording symptoms: liver-gallbladder dampness-heat syndrome (LGDHS, *n* = 7) and liver-kidney Yin deficiency syndrome (LKYDS, *n* = 10) [69]. The TCM ZHENG types were identified by three chief or deputy physicians according to “evaluation criteria of the clinical diagnosis, drug efficacy and ZHENG classification for cirrhosis (pilot program)” [70]. This classification was then verified by metabonomics. (Figure 1).

**Figure 2:**
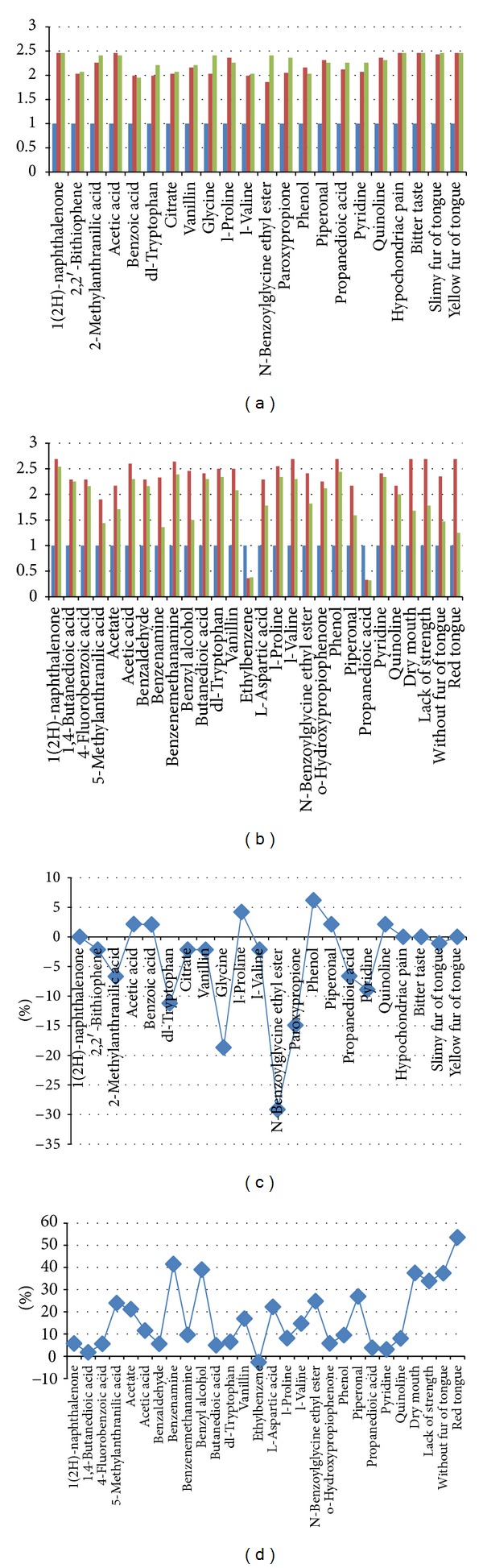
The reversion maps reveal the therapeutic effects of two TCM ZHENG types in hepatitis-B-caused cirrhosis. (a) and (b) Histogram of the therapeutic effects of FZHY Tablet for liver-gallbladder dampness-heat syndrome and liver-kidney Yin deficiency syndrome, respectively, by the changing trend of significantly differential metabolites and classic symptoms. Blue, red, and green bars stand for control group, before intervention and 12-week intervention of FZHY tablet. The *x*-axis represents the changed metabolites and symptoms, and the *y*-axis is fold change of mean ranks calculated by the Mann-Whitney test, compared with the control group. (c) and (d) Curve diagram of regression trend of significantly differential metabolites and classic symptoms in liver-gallbladder dampness-heat syndrome and liver-kidney Yin deficiency syndrome respectively, for 12-week intervention of FZHY tablet. The *x*-axis represents the changed metabolites and symptoms, and the *y*-axis is the rate of reversion, representing the therapeutic effect of FZHY tablet for different ZHENGs.

Furthermore, there was a significant difference in metabolites between CG and two ZHENGs which were selected by OPLS loading plot analysis. The differentiated metabolites combined with classic symptoms and feelings (bitter taste, hypochondriac pain, slimy fur of tongue, and yellow fur of tongue in LGDHS, while dry mouth, lack of strength, without fur of tongue, and red tongue in LKYDS) were chosen as the potential markers for each ZHENG. The reversions of these metabolites and hierarchical corresponding symptoms were used as indicators of the therapeutic effect of FZHY tablets and found that FZHY tablets are more effective for LKYDS than for LGDHS, which coincides with the treatment principles of TCM (Figure 2). This section relates to the FZHY study only. Further details are provided in the Supplementary Methods available online at http://dx.doi.org/10.1155/2013/235969.

Because every “-Omics” has the similar features in Systems Biology, as holistic aspects, noninvasive, integrity, multi-target, high-throughput and digitalization, the similar thought could be applied to other “-Omics.” It is to say that gene sequences or proteins could be combined with clinical information to provide the NET-Marker of the special ZHENG. Thus, our initial exploration provided strong evidence of the feasibility and robustness of ZHENG-Omics, even though we did not obtain a full validation.

## 5. Discussion and Prospect

The outcome of our exploration is inspiriting us to assess the curative effect based on ZCT by ZHENG-omics that combines omic data with clinical symptoms and feelings. We hope this approach will narrow the gap between mainstream medicine and TCM.

Compared with pharmaco-omics [1] and other “-Omics,” ZHENG-omics may show some promise as a new method having distinct advantages. First of all, ZHENG-omics may give the guide to pharmaco-omic performance with the clinical experience of 3000 years based on ZCT, though lacking enough evident ascertainments. The ZHENG classification may guide patient classification of predose phenotype and prescribe drugs according to the phenotype. Furthermore, ZHENG-Omics could provide the profound connotation of the disease, which consists of not only genetic and environmental factors but also emotional and spiritual factors. This notion resembles the new treatment concept of modern medicine that a treatment should not only relieve symptoms but should also take care of the mental health of a patient. In this respect, ZHENG-omics may provide some new revelations for personalized medicine.

However, every coin has two sides, so as to current “Omics.” Every “Omic” has the advantages and disadvantages (Table 3). If using only one “Omics” for study of ZHENG, the shortcomings such as deficiency of cross-reference and scientific discrimination, overloading information, and excessive simplification will show up. On the other side, one can carry out ZHENG-omics at different levels in multiple research centers. Such research can find those specific and meaningful markers distinguishing primary factors from secondary factors. With the multidimensional studies of DNA, proteins, and metabolites, the variability, integrity, and complexity of TCM ZHENGs will be annotated commendably and can be used as basis for understanding and differentiating them. Furthermore, we should combine microscopic omic data with macroscopic symptoms, with the methods such as association rules [71] and bioinformatics [72]. Yet, for ZHENG-omics study, one should pay special attention to the following aspects.

First of all, careful screening and data collection of samples from patients with the most classic ZHENGs are necessary. Before this can be initialized, the patients with classic ZHENG should be targeted, according to the most typical syndromes getting out from literature retrieval and data mining of a lot of clinical cases [73, 74]. Patient samples for a special ZHENG can be found based on these typical symptoms. The more samples that are collected, the more precision will be developed to the essence of ZHENG, leading to more accurate and objective classification. 

Second, the existing strategy should be refined. Since one ZHENG (one syndrome) can change to another ZHENG dynamically, the current static study cannot meet the needs of deep research and clinical application. Luckily, ZHENG-omics provides a high-throughput and noninvasive clinical research tool and enables study on the dynamical ZHENG classification in an extending period. Particularly, we advocate that initiative and passive intervention could be used to study development and dynamic Classification of ZHNEG, with the help of tracer techniques such as fluorescence labeling. Nowadays, most attention is paid to preliminary intervention by inducers [1, 21] and environmental factors [75], while purposeful, further continuous intervention will provide deeper vision into the regularity of ZHENG classification.

Since ZHENGs are frequently considered as phenotypes of a disease, researchers are mostly focused on the different ZHENGs of the same disease. However, we should pay more attention to the formerly mentioned phenomenon that the same syndrome appears in different diseases. This part will bring another look to old drugs as treatments for other diseases.

## 6. Conclusion

**Figure 3:**
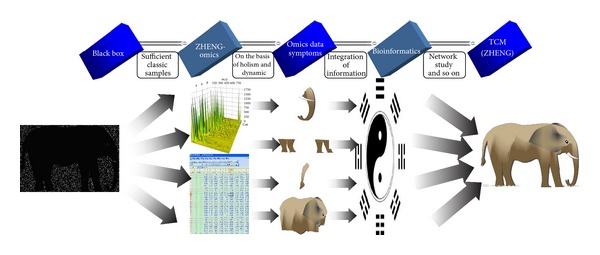
Schematic diagram of research approach for ZHENG-omics. Traditional Chinese medicine is mysterious and obscure, like a fuzzy picture. It is difficult to obtain systematic knowledge from the whole directly, yet the partial information is much easier to be acquired and understood, such as nucleic acids, proteins, and metabolites. Combined with classic symptoms and feelings, the former could be obtained by ZHENG-omics from sufficient classic samples on the basis of holism. The integration of omic data and symptoms will be performed by bioinformatics and so on, which further provide the multilevel information, like small lights in the big black box. Then, a vivid picture may emerge in our sights, just like getting the systematic connotation of TCM or ZHENG.

Just like a blindfold boy will feel an elephant, snake, tree, wall, and rope would be in his mind when he touched the squirming trunk, grossus knee, broad body, and swinging tail, respectively. He may perceive the whole elephant from combination of these incomplete parts. Huge and metaphysical TCM, especially ZHENG, is corresponding to the elephant, while ZHENG-omics will provide the multilevel information, just like omic data and symptoms compared to tusk, knee, and tail. It is important to note that dynamic and intervention will also paint a vivid picture (Figure 3). Under the strategy of “black box-partial system-whole system,” the connotation of systematic TCM will emerge in our sights.

Furthermore, the ZHENG-omics possesses and integrates the characteristics of both ZHENG and systems biology and may advance TCM to a new level. It would overcome the shortcomings of current methods for evaluating personal diagnosis, complex intervention, and patients' feelings. Through ZHENG-omic study, personal medicine in TCM may emerge eventually and may change the current medical system. We believe that the advantage of TCM in theory and experience will overcome the limitations of current personal medicine and contribute to the overall world healthcare.

## Supplementary Material

Supplemental Figure 1: Schematic diagram of research approach for ZHENG-Omics.
Supplemental Figure 2: OPLS score plots of metabolic profiles.
Supplementary Table 1: Demographics of the patients with liver-gallbladder dampness-heat syndrome (LGDHS) and liver-kidney yin deficiency syndrome (LKYDS).
Supplemental Table 2: Summary of the Modeling Quality of OPLS.
Supplemental Table 3: OPLS score plots of metabolic profiles.
Supplemental Table 4: Brief information of Markers of liver-kidney yin deficiency syndrome.

## Figures and Tables

**Table 1:** Clinical researches of TCM ZHENGs with systems biology.

Syndromes	Disease	Researchers	Omics
Liver-gallbladder dampness-heat	Hypertension	Chu et al. [22]	Proteomics
	Chronic hepatitis B	Guo et al. [23]	

Liver Yang transforming into wind	Cerebral infarction	Zeng et al. [24]	Proteomics

Liver stagnation	Depression, premenstrual syndrome, menopausal syndrome	Tan et al. [25]	Proteomics

	Rheumatoid arthritis	Lu et al. [26]	Genomics
Cold and heat pattern	Cold-syndrome genealogy	Wang et al. [27]	Genomics
	Rheumatoid arthritis	Gu et al. [28]	Metabolomics

	Colorectal cancer	Yang et al. [29]	Genomics
Spleen deficiency	Chronic superficial gastritis	Yang et al. [30]	Genomics
	Gastritis	Liu et al. [31]	Proteomics

	Diabetes	Weng et al. [32]	Genomics
		Wu et al. [11]	Genomics
Kidney deficiency	Normal	Ni et al. [12]	Genomics
	Normal	Liu et al. [14]	Proteomics
	Chronic heart failure	Zheng et al. [33]	Metabolomics

Dampness syndrome	Chronic hepatitis B	Guan et al. [34]	Genomics
	Chronic gastritis	Wang et al. [35]	Proteomics

Dampness-phlegm	Obesity	Wang et al. [36]	Genomics

	Coronary heart disease	Ma et al. [37]	Genomics
	Coronary heart disease	Yuan et al. [38]	Genomics
Blood stasis	Coronary heart disease	Yuan et al. [39]	Genomics
	Coronary heart disease	Wu et al. [40]	Proteomics
	Unstable angina	Wang et al. [41]	Metabolomics

	Hepatitis B cirrhosis	Li et al. [42]	Genomics
	Chronic hepatitis B	Song et al. [43]	Proteomics
Deficiency syndrome	Primary liver cancer	Chen et al. [44]	Metabolomics
	Diabetes mellitus	Wu et al. [45]	Metabolomics
	Hypertension	Yang et al. [46]	Metabolomics

Liver-kidney Yin deficiency syndrome	Hepatocellular carcinoma	Weng et al. [47]	Genomics

Ascendant hyperactivity of Ganyang syndrome	Hypertension	Jiang et al. [48]	Metabolomics

**Table 2:** Experimental researches of TCM ZHENGs with systems biology.

Syndromes	Disease	Researchers	Omics
Ascendant hyperactivity of liver Yang	Migraine headache	Hu et al. [49]	Proteomics
	Migraine headache	Zhong et al. [50]	Proteomics
	Hypertension	Zhou et al. [51]	Proteomics
	Hypertension	Zhang et al. [52]	Proteomics

Liver depression		Zhong et al. [53]	Proteomics

Spleen deficiency		Wang et al. [54]	Proteomics
		Luo et al. [55]	Metabolomics

		Shen et al. [56]	Genomics
		Tang et al. [15]	Proteomics
Kidney deficiency	Chronic heart failure	Zheng et al. [33]	Metabolomics
			Lu et al. [17]
		Wang et al. [16]	Metabolomics
		Chen et al. [57]	Metabonomics

Blood stasis-phlegm	Coronary heart disease	Liu et al. [58]	Proteomics

Blood deficiency		Tong et al. [59]	Genomics

Blood stasis	Coronary heart disease	Jian et al. [60]	Metabolomics
	Coronary heart disease	Wang et al. [61]	Metabolomics

Yang Qi deficiency	H22 tumor-bearing	Pan et al. [62]	Genomics

**Table 3:** Brief introduction of  “-omics” and bioinformatics.

Omics	Advantages	Disadvantages	Literatures
Genomics (transcriptomics)	Gene polymorphism Susceptibility for prognosis and treatment Completed database High throughput	Nonassociation to regulation of life activities Nonconsistent strictly with mRNA expression	Wu et al. [11] Lu et al. [63]
Proteomics	Performer of life function	Instability Variability	Liu et al. [64] Lu et al. [65]
Metabonomics	Amplified action Simplicity to detect Less numbers Similarities in different species	Lack of beneficial supports Interferences by physiological factors	van Wietmarschen et al. [66] Sun et al. [67] Liu et al. [31]
Bioinformatics	Totally holism Exploration the potential of information, Focusing on function relation	Needing of self-development	Li et al. [18]
