# Supplementary material for: ZHENG-Omics Application in ZHENG Classification and Treatment: Chinese Personalized Medicine
Source: Evid Based Complement Alternat Med. 2013 Apr 3;2013:235969. doi: 10.1155/2013/235969 (PMC3638582; doi:10.1155/2013/235969)
Supplement: Supplementary file 1 — Supplemental Figure 1: Schematic diagram of research approach for ZHENG-Omics. Supplemental Figure 2: OPLS score plots of metabolic profiles. Supplementary Table 1: Demographics of the patients with liver-gallbladder dampness-heat syndrome (LGDHS) and liver-kidney yin deficiency syndrome (LKYDS). Supplemental Table 2: Summary of the Modeling Quality of OPLS. Supplemental Table 3: OPLS score plots of metabolic profiles. Supplemental Table 4: Brief information of Markers of liver-kidney yin deficiency syndrome. [file 235969.f1.doc]

**Supplementary information**

**Supplementary Figure 1**

**
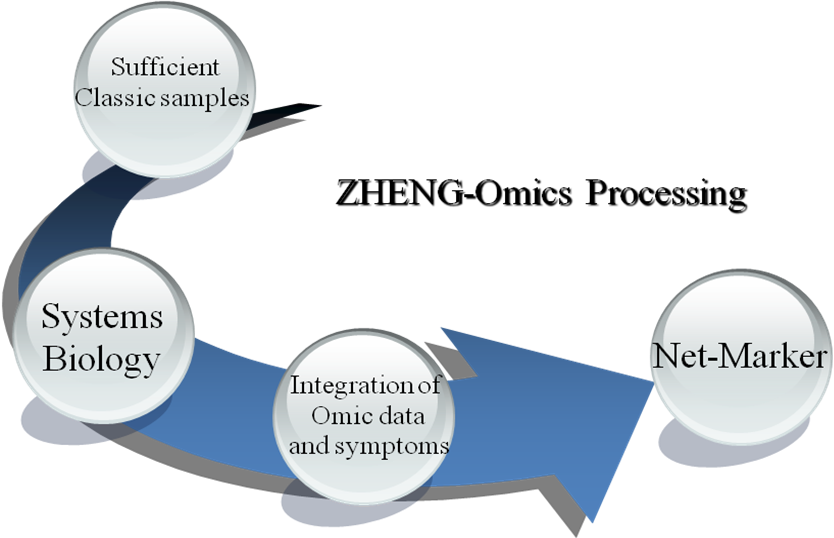
**

[**Schematic diagram**](app:ds:schematic diagram) **of research approach for ZHENG-Omics. The scope of ZHENG-Omics can be illustrated as follows. Firstly, clinical symptoms are classified into several groups according to the principle of ZHENG Differentiation, and the difference in biological markers, including DNA, RNA, proteins and metabolites, are identified among these groups. Secondly, integrating of genes, proteins, metabolites, symptoms and others, NET-Markers of ZHENG will be obtained from former differences by bioinformatics and other mathematical analysis. The markers could then be used to provide the basis in developing a possible population-screening tool for selecting target-individuals and creating evaluation index for personalized treatment based on ZHENG Differentiation. Finally, ZHENG-Omics will give an objective and practical evaluation to the classical “ZHENG Differentiation and Treatment”.**

**Supplementary Figure 2**

**
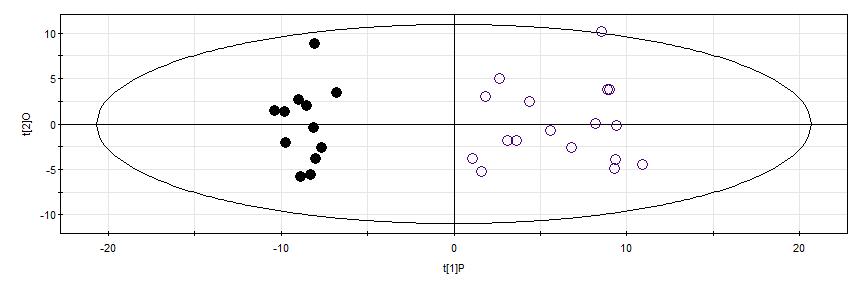
**

a

**
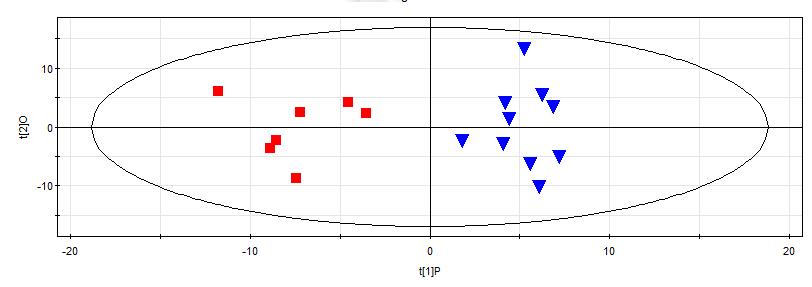
**

b


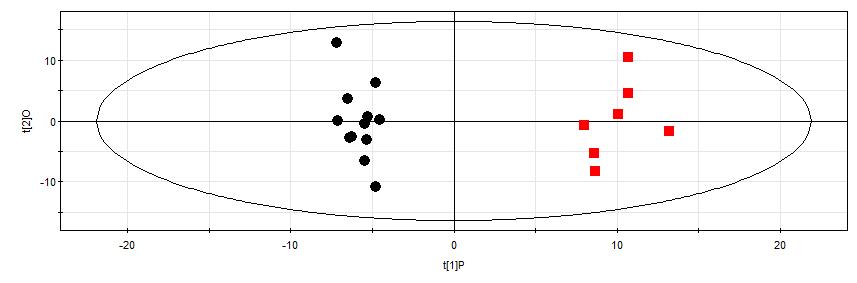

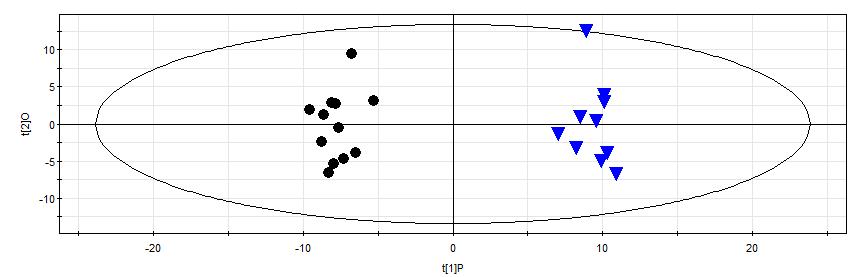


c

d

**OPLS score plots of metabolic profiles. Black dot, purple circle, red box and blue triangle represent Control Group (CG), Hepatitis B caused Cirrhosis Group (HBC), liver-gallbladder dampness-heat syndrome Group (LGDHS) and liver-kidney yin deficiency syndrome Group (LKYDS) respectively. OPLS score plot of metabolic profiles of GC-MS between the CG and the HBC (a), the LGDHS group and the LKYDS (b), the CG and the LGDHS (c), and the CG and the LKYDS (d).**

**Supplementary Methods**

- 1. *Subjects and sample collection*

The FZHY study was performed in accordance with the principles contained in the Declaration of Helsinki and was approved by the local ethics committee. All of the subjects in the study were recruited from Shanghai Shuguang hospital (Shanghai, China) and were given informed consents. 17 patients with Hepatitis B caused Cirrhosis (HBC) were enrolled as the HBC group and further 12 healthy subjects were recruited as control group. The information of all subjects is summarized in Supplementary Table 1. Diagnosis standard of cirrhosis referred to "The guidelines of prevention and treatment for chronic hepatitis B". And all cases of HBC caused by other factors such as hepatitis C infection, alcohol consumption, usage of drugs with hepatotoxicity, were ruled out before all the subjects entered the study. Among the enrolled patients, 7 were diagnosed as liver-gallbladder dampness-heat syndrome (LGDHS) and the other 10 as liver-kidney yin deficiency syndrome (LKYDS) by three chief or deputy physicians, according to "evaluation criteria of the clinical diagnosis, drug efficacy and ZHENG differentiation for cirrhosis (pilot program)". The classic symptoms were gradated to 5 degrees and recorded. The HBC subjects were treated with a single oral dose of FZHY tablets (0.08g/kg/day) Urina sanguinis of 12 healthy subjects and the pre-dose and post-dose (12 weeks) Urina sanguinis of 17 HBC patients were collected and stored at −80°C until GC-MS assay.

- 1. *Chemicals and drugs*

Ethyl chloroformate (ECF), pyridine, anhydrous ethanol, sodium hydroxide, chloroform, and anhydrous sodium sulfate were analytical grade from China National Pharmaceutical Group Corporation (Shanghai, China). L-2-chlorophenylalanine (Shanghai Intechem Tech. Co. Ltd., China) was used as an internal quality standard which was prepared in the ultrapure water from a Milli-Q system (Millipore, USA). FZHY tablets were provided by Shanghai Huanghai Pharmaceutical Co., Ltd.

- 1. *Sample preparation*

All these samples were thawed in ice water bath and vortex-mixed before analysis. Each 600μL aliquot of standard mixture or urine sample was added to a screw tube. After adding 100μL of l-2-chlorophenylalanine (0.1mg×mL^−1^), 400μL of anhydrous ethanol, and 100μL of pyridine to the urine sample, 50 μL of ECF was added for first derivatization at 20.0±0.1°C. The pooled mixtures were sonicated at 40 kHz for 60 s. Subsequently, extraction was performed using 300μL of chloroform, with the aqueous layer pH carefully adjusted to 9-10 using 100μL of NaOH (7mol×L^−1^). The derivatization procedure was repeated with the addition of 50μL ECF into the aforementioned products. After the two successive derivatization steps, the overall mixtures were vortexed for 30 s and centrifuged for 3min at 3000 rpm. The aqueous layer was aspirated off, while the remaining chloroform layer containing derivatives were isolated and dried with anhydrous sodium sulfate and subsequently subjected to GC–MS. The derivatization method referred to the reference.

- 1. *Data acquisition*

All GC–MS analyses were performed by a mass spectrometer 5975B (Agilent technologies, USA) coupled with an Agilent 6890 (Agilent technologies, USA) gas chromatography instrument. In the gas chromatographic system, a catabletary column (Agilent J&W DB-5ms Ultra Inert 30m×0.25mm, film thickness 0.25μm) was used. Helium carrier gas was used at a constant flow rate of 1.0 ml×min^-1^. 1.0μl of derivatized samples was injected into the GC/MS instrument, and splitless injection mode was used. To acquire a well separation, the column temperature was initially maintained at 80°C for 2 min, and then increased from 80 to 140°C at the rate of 10°C/min for 6 min. Then, the column temperature was increased to 240°C at the rate of 4°C/min for 25min. After that, the column temperature was increased to 280°C at the rate of 10°C/min for 4 min and held for 3 min. The temperatures of the injection port, the interface and source temperature were set at 280°C, 260°C and 230°C, respectively. The measurements were made with electron impact ionization (70 eV) in the full scan mode (m/z 30–550). The solvent post time was set to 5 min.

- 1. *Data analysis*

After the total ion current chromatograms (TICs) were obtained, peak-alignment or warping techniques are commonly applied to compensate for minor shifts in retention times caused by experimental variations and column aging. All the GC–MS raw files were converted to CDF format via the software come with Agilent MSD workstation and were subsequently processed by the XCMS toolbox (<http://metlin.scripps.edu/download/>) using the default settings with the following exceptions: xcmsSet (full width at half-maximum: fwhm=5; S/N cutoff value: snthresh =10, max =15), group (bw =5). The resulting table (CSV file) was exported into Microsoft Excel (Microsoft Inc., USA), where normalization was performed prior to multivariate analyses.

Then the resulting three dimensional matrix involving peak index (RT-m/z pair), sample names (observations), and normalized peak area percent was introduced into Simca-P 12.0 Software package (Umetrics, Umea, Sweden) for partial least squares-discriminate analysis (PLS-DA) for three groups and orthogonal partial least square (OPLS) for each two groups. The healthy and two ZHENGs were distinguished by OPLS analysis respectively. The model information is shown in Supplementary Table 2, and two maps of OPLS score plot are presented in Supplementary Figure 2. Differential metabolites between CG and HBCG were generated by OPLS loadings plot by VIP (variable influence on projection; >1.5) value (Supplementary Figure 2). VIP values characterize the relative overall importance of the individual variables to the model. In the second step, those variables were compared by Mann-Whitney U-test to confirm the changed metabolites in SPSS 17.0 (SPSS, Chicago, IL, USA) with the threshold of P-value set at 0.05(two-tailed). Those variables, then, could be identified by standards or searching in NIST 2005 database. The differential metabolites’ information is shown in Supplementary Table 3, 4.

The potential Net-Marker was obtained from the integration of differential metabolites, hierarchical corresponding symptoms and feelings. These differences of the pre-dose and post-dose patients were further compared with healthy subjects by Mann-Whitney U-test in average rank sum test. The evaluation of ZHENG Differentiation and Treatment were obtained by reversions of the Net-Marker. (Supplementary Table 3, 4)

**Supplementary Table 1**

**Demographics of the patients with liver-gallbladder dampness-heat syndrome (LGDHS) and liver-kidney yin deficiency syndrome (LKYDS)**

| Parameter | LGDHS (n=7) | LKYDS(n=10) | P-Value |
| --- | --- | --- | --- |
| Age (years) | 53.10±8.30 | 56.60±12.01 | 0.52 |
| Sex(male/female) | 4/3 | 7/3 | 0.61 |
| Weight(kg) | 66.86±8.80 | 62.80±8.00 | 0.35 |
| Child-Pugh Score | 7.57±1.81 | 6.40±1.35 | 0.17 |
| ALT | 35.86±17.04 | 33.00±27.90 | 0.81 |
| GGT | 55.43±33.40 | 83.50±122.40 | 0.57 |

**The Child-Pugh score and the content of alanine transarninase (ALT) were slightly higher in LGDHS than in LKYSD and the content of glutamyl transpeptidase(GGT) was slightly more in LKYSD than in LGDHS. However, these differences were not significant (p>0.05, T-test, two-tailed) in this study.**

**Supplementary Table 2**

**Summary of the Modeling Quality of OPLS**

| Group | No^a^ | R2Xcum^b^ | R2Ycum^c^ | Q2Ycum^d^ |
| --- | --- | --- | --- | --- |
| CHB versus CG | 1P + 1O e | 0.230 | 0.956 | 0.751 |
| LKYDS versus LGDHS | 1P + 1O e | 0.261 | 0.904 | 0.427 |
| LKYDS versus CG | 1P + 1O e | 0.274 | 0.975 | 0.844 |
| LGDHS versus CG | 1P + 1O e | 0.115 | 0.984 | 0.859 |

**^a^ No represents the number of components.**

**^b, c^ R^2^X_cum_ and R^2^Y_cum_ represent the cumulative Sum of Squares (SS) of all the X’s and Y’s explained by all extracted components.**

**^d^ Q^2^Y_cum_ is an estimate of how well the model predicts the Y’s.**

**^e^1P+1O, one predictive component and one orthogonal component for establishing the orthogonal partial least square (OPLS) model.**

**Supplementary Table 3**

**Brief information of Markers of liver-gallbladder dampness-heat syndrome**

| Markers | P-Value^a^ | VIP^b^ | FC^c^ | | R^d^ |
| --- | --- | --- | --- | --- | --- |
|  |  |  | 0W | 12W |  |
| 1(2H)-Naphthalenone | 0.000 | 2.16 | 2.46 | 2.46 | 0.00% |
| 2,2'-Bithiophene | 0.004 | 1.69 | 2.03 | 2.07 | -2.14% |
| 2-Methylanthranilic acid | 0.000 | 1.98 | 2.26 | 2.41 | -6.68% |
| Acetic acid | 0.000 | 2.22 | 2.46 | 2.41 | 2.15% |
| Benzoic acid | 0.005 | 1.55 | 1.99 | 1.95 | 2.09% |
| dl-Tryptophan | 0.005 | 1.56 | 1.99 | 2.21 | -11.22% |
| Citrate | 0.004 | 1.55 | 2.03 | 2.07 | -2.14% |
| Vanillin | 0.001 | 1.84 | 2.16 | 2.21 | -2.16% |
| Glycine | 0.004 | 1.54 | 2.03 | 2.41 | -18.68% |
| l-Proline | 0.000 | 1.78 | 2.36 | 2.26 | 4.21% |
| l-Valine | 0.005 | 1.55 | 1.99 | 2.03 | -2.14% |
| N-Benzoylglycine ethyl ester | 0.013 | 1.52 | 1.86 | 2.41 | -29.15% |
| Paroxypropione | 0.003 | 1.64 | 2.05 | 2.36 | -14.92% |
| Phenol | 0.001 | 1.72 | 2.16 | 2.03 | 6.18% |
| Piperonal | 0.000 | 1.60 | 2.31 | 2.26 | 2.13% |
| Propanedioic acid | 0.002 | 1.74 | 2.12 | 2.26 | -6.62% |
| Pyridine | 0.003 | 1.67 | 2.07 | 2.26 | -8.91% |
| Quinoline | 0.000 | 2.00 | 2.36 | 2.31 | 2.13% |
| hypochondriac pain | 0.000 | 2.41 | 2.46 | 2.46 | 0.00% |
| bitter taste | 0.000 | 2.30 | 2.46 | 2.46 | 0.00% |
| slimy fur of tongue | 0.000 | 2.21 | 2.43 | 2.46 | -1.09% |
| yellow fur of tongue | 0.000 | 2.26 | 2.46 | 2.46 | 0.00% |

**^a^ P-Value was obtained from Mann-Whitney test(two-tailed).**

**^b^ VIP, variable importance in the project**

**^c^ FC is fold change of mean ranks calculated by the Mann-Whitney test. 0W: pre-dose; 12W: post-dose**

**^d^ R: Rate of reversion= (FC_0W_-FC_12W_)/FC_0W_**

**Supplementary Table 4**

**Brief information of Markers of liver-kidney yin deficiency syndrome**

| Markers | VIP^a^ | P-Value^b^ | FC^c^ | | R^d^ |
| --- | --- | --- | --- | --- | --- |
|  |  |  | 0W | 12W |  |
| 1(2H)-Naphthalenone | 1.93 | 0.000 | 2.69 | 2.54 | 5.71% |
| 1,4-Butanedioic acid | 1.50 | 0.000 | 2.29 | 2.25 | 1.73% |
| 4-Fluorobenzoic acid | 1.56 | 0.000 | 2.29 | 2.16 | 5.54% |
| 5-Methylanthranilic acid | 1.51 | 0.007 | 1.90 | 1.44 | 23.92% |
| Acetate | 1.53 | 0.001 | 2.17 | 1.71 | 21.12% |
| Acetic acid | 1.53 | 0.000 | 2.60 | 2.30 | 11.55% |
| Benzaldehyde | 1.51 | 0.000 | 2.29 | 2.16 | 5.54% |
| Benzenamine | 1.59 | 0.000 | 2.33 | 1.36 | 41.48% |
| Benzenemethanamine | 1.51 | 0.000 | 2.64 | 2.39 | 9.62% |
| Benzyl alcohol | 1.83 | 0.000 | 2.46 | 1.50 | 38.97% |
| Butanedioic acid | 1.63 | 0.000 | 2.41 | 2.30 | 4.93% |
| dl-Tryptophan | 1.66 | 0.000 | 2.50 | 2.34 | 6.43% |
| Vanillin | 1.80 | 0.000 | 2.50 | 2.08 | 16.90% |
| Ethylbenzene | 1.63 | 0.000 | 0.36 | 0.38 | -2.75% |
| L-Aspartic acid | 1.54 | 0.000 | 2.29 | 1.78 | 22.20% |
| l-Proline | 1.66 | 0.000 | 2.55 | 2.34 | 8.11% |
| l-Valine | 1.88 | 0.000 | 2.69 | 2.30 | 14.75% |
| N-Benzoylglycine ethyl ester | 1.70 | 0.000 | 2.41 | 1.82 | 24.79% |
| o-Hydroxypropiophenone | 1.55 | 0.000 | 2.25 | 2.12 | 5.75% |
| Phenol | 1.95 | 0.000 | 2.69 | 2.44 | 9.46% |
| Piperonal | 1.51 | 0.000 | 2.17 | 1.59 | 26.92% |
| Propanedioic acid | 1.54 | 0.000 | 0.33 | 0.32 | 3.76% |
| Pyridine | 1.58 | 0.001 | 2.41 | 2.34 | 3.02% |
| Quinoline | 1.54 | 0.000 | 2.17 | 2.00 | 7.98% |
| dry mouth | 2.08 | 0.000 | 2.69 | 1.68 | 37.53% |
| lack of strength | 2.15 | 0.001 | 2.69 | 1.78 | 33.84% |
| without fur of tongue | 1.84 | 0.000 | 2.35 | 1.47 | 37.40% |
| red tongue | 1.99 | 0.000 | 2.69 | 1.25 | 53.53% |

**^a^ P-Value was obtained from Mann-Whitney test(two-tailed).**

**^b^ VIP, variable importance in the project**

**^c^ FC is fold change of mean ranks calculated by the Mann-Whitney test. 0W: pre-dose; 12W: post-dose**

**^d^ R: Rate of reversion= (FC_0W_-FC_12W_)/FC_0W_**
